# A generalized approach to predicting protein-protein interactions between virus and host

**DOI:** 10.1186/s12864-018-4924-2

**Published:** 2018-08-13

**Authors:** Xiang Zhou, Byungkyu Park, Daesik Choi, Kyungsook Han

**Affiliations:** Department of Computer Engineering, Inha University, Incheon, 22212 South Korea

**Keywords:** Virus and host, Interspecies protein-protein interaction, Prediction model

## Abstract

**Background:**

Viral infection involves a large number of protein-protein interactions (PPIs) between virus and its host. These interactions range from the initial binding of viral coat proteins to host membrane receptor to the hijacking the host transcription machinery by viral proteins. Therefore, identifying PPIs between virus and its host helps understand the mechanism of viral infections and design antiviral drugs. Many computational methods have been developed to predict PPIs, but most of them are intended for PPIs within a species rather than PPIs across different species such as PPIs between virus and host.

**Results:**

In this study, we developed a prediction model of virus-host PPIs, which is applicable to new viruses and hosts. We tested the prediction model on independent datasets of virus-host PPIs, which were not used in training the model. Despite a low sequence similarity between proteins in training datasets and target proteins in test datasets, the prediction model showed a high performance comparable to the best performance of other methods for single virus-host PPIs.

**Conclusions:**

Our method will be particularly useful to find PPIs between host and new viruses for which little information is available. The program and support data are available at http://bclab.inha.ac.kr/VirusHostPPI.

## Background

There are many types of viruses that cause a wide variety of viral infections or viral diseases. For example, more than 11,000 deaths were reported in Africa during the outbreak of Ebola virus disease in 2014 and 2015 [1]. More recently, an outbreak of Middle East respiratory syndrome coronavirus (MERS-CoV) [2], which began with a patient in an emergency room, occurred in South Korea. So far, there is no specific vaccine or effective treatment for Ebola virus and MERS-CoV [1, 2]. Viral infection involves a large number of protein-protein interactions (PPIs) between virus and its host. These interactions range from the initial binding of viral coat proteins to host membrane receptor to the hijacking the host transcription machinery by viral proteins. Therefore, identifying PPIs between virus and its host helps understand the mechanism of viral infections and design antiviral drugs.

Many computational methods have been developed to predict PPIs, but most of them are intended for PPIs within a same species rather than for PPIs across different species. Methods for predicting intra-species PPIs do not distinguish interactions between proteins of the same species from those of different species, and thus are not appropriate for predicting inter-species PPIs. Motivated by a recent increase in data of virus-host PPIs, a few computational methods have been developed to predict virus-host PPIs using machine learning methods. For instance, a homology-based method [3] and domain-based method [4] were proposed to predict PPIs between *H. sapiens* and *M. tuberculosis* H37Rv. Cui et al. [5] developed a support vector machine (SVM) model to predict PPIs between human and two types of viruses (hepatitis C virus and human papillomavirus). However, these prediction methods cannot be applied to new viruses or new hosts that have no known PPIs to the methods. Inter-species PPIs predicted by these methods are for PPIs between virus of a single type and host of a single type. A recent SVM model called DeNovo is perhaps the only one that can predict PPIs of new viruses with a shared host [6]. Amino acid sequence similarity between different types of viruses or hosts is relatively low, so sequence-based prediction of virus-host PPIs for new viruses or hosts is quite challenging. In this study, we developed a new prediction method of virus-host PPIs which is applicable to new viruses or hosts. The rest of this paper discusses the details of the method and its experimental results.

## Methods

### Data of virus-host PPIs

We obtained all known PPIs between virus and host using the PSICQUIC web service (http://www.ebi.ac.uk/Tools/webservices/psicquic/view/main.xhtml). We extracted virus-host PPIs from four databases, APID, IntAct, Mentha and UniProt, which use same protein identifiers. The sequences of the proteins involved in any of the PPIs were obtained from the UniProt database (http://www.uniprot.org). As of December 2016, there are a total of 12,157 PPIs between 29 hosts and 332 viruses (Table 1). The reason that human is listed as a separate category from other animals (i.e., non-human animals) in the classification of hosts is because human has a much larger number of known PPIs with viruses than other animals. Detailed information on the viruses involved in the virus-host PPIs is available at http://bclab.inha.ac.kr/VirusHostPPI.

**Table 1:** The number of known host–virus PPIs and viruses interacting with a host

Host	Major hosts	#Host-virus	#Interacting
classification	(taxonomy ID)	PPIs	virus taxanomy IDs
Human	Homo sapiens (9606)	11,491	246
	Mus musculus (10090)	191	89
	Bos taurus (9913)	125	32
	Rattus norvegicus (10116)	86	19
Non-human	Sus scrofa (9823)	57	10
animal	Gallus gallus (9031)	15	9
	Equus caballus (9796)	7	6
	Drosophila melanogaster (7227)	4	3
	Canis lupus familiaris (9615)	3	1
Plant	Arabidopsis thaliana (3702)	17	11
	Escherichia coli K-12 (83333)	78	9
Bacteria	Streptococcus pneumonia (170187)	49	2
	Pseudomonas aeruginosa (208963)	13	4
	Escherichia coli (562)	3	1
Others	15 hosts	18	15
Total	29	12,157	332 ^∗^

332*: the total number of non-redundant viruses in terms of taxonomy IDs

Learning-based prediction of PPIs requires both positive and negative PPI data, but negative data are not readily available in databases. For negative data, we obtained protein sequences of major hosts (human, non-human animal, plant, and bacteria) from UniProt, and removed those with a sequence similarity higher than 80% to any positive data using CD-HIT-2D [7].

### Datasets

We constructed several datasets to examine the applicability of our prediction method to new viruses or hosts. The datasets are classified into two types:

1. Training (TR) and test (TS) sets for assessing the applicability to new viruses TR1: PPIs between human and any virus except H1N1 TR2: PPIs between human and any virus except Ebola virus TR3: PPIs between any host and any virus except H1N1 TR4: PPIs between any host and any virus except Ebola virus TS1: PPIs between human and H1N1 virus TS2: PPIs between human and Ebola virus
2. Training (TR) and test (TS) sets for assessing the applicability to new hosts TR5: PPIs between human and any virus TS5.1: PPIs between non-human animal and any virus TS5.2: PPIs between plant and any virus TS5.3: PPIs between bacteria and any virus TS5.4: PPIs between any non-human host and any virus

**Fig. 1:**
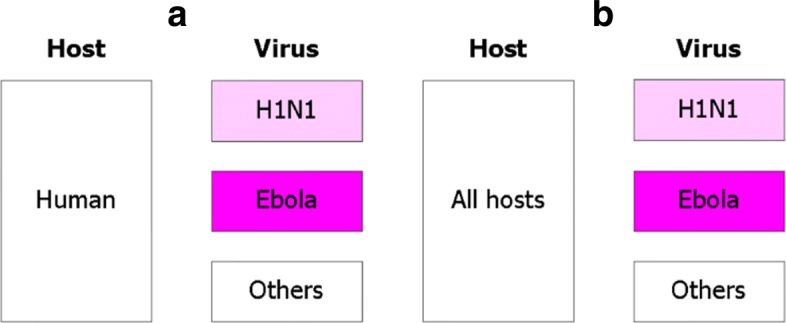
**a** Training dataset 1 (TR1): 10,955 PPIs between human and any virus except H1N1. Test dataset 1 (TS1): 381 PPIs between human and H1N1 virus. Training dataset 2 (TR2): 11,341 PPIs between human and any virus except Ebola virus. Test dataset 2 (TS2): 150 PPIs between human and Ebola virus. **b** Training dataset 3 (TR3): 11,617 PPIs between any host and any virus except H1N1. Test dataset 1 (TS1): 381 PPIs between human and H1N1 virus. Training dataset 4 (TR4): 12,007 PPIs between any host and any virus except Ebola virus. Test dataset 2 (TS2): 150 PPIs between human and Ebola virus

To examine the applicability of the prediction method to new viruses, we constructed a training dataset with 10,955 PPIs between human and any virus except H1N1 virus (hereafter called TR1). The prediction method was later tested on a test dataset with 381 PPIs between human and H1N1 virus (called TS1), which were not used in training the method. We constructed another training dataset TR2 with 11,341 PPIs between human and any virus except Ebola virus. The prediction method trained with TR2 was tested on a test dataset TS2, which contains 150 PPIs between human and Ebola virus (Fig. 1a). Additional training datasets for studying the applicability to new viruses are TR3 and TR4. TR3 contains 11,617 virus-host PPIs except PPIs of H1N1 virus. TR4 consists of 12,007 virus-host PPIs except PPIs of Ebola virus. The prediction model trained with TR3 and TR4 was later tested on TS1 and TS2, respectively (see Fig. 1b for details).

The reason for selecting the viruses for the SVM model is as follows: (1) For training the SVM model, we tried to select as many virus proteins as possible which have known interactions with host proteins. (2) For testing the SVM method on new viruses, we selected H1N1 and Ebola virus because the viruses caused a large number of deaths recently but no specific vaccine or effective treatment is available yet.

**Fig. 2:**
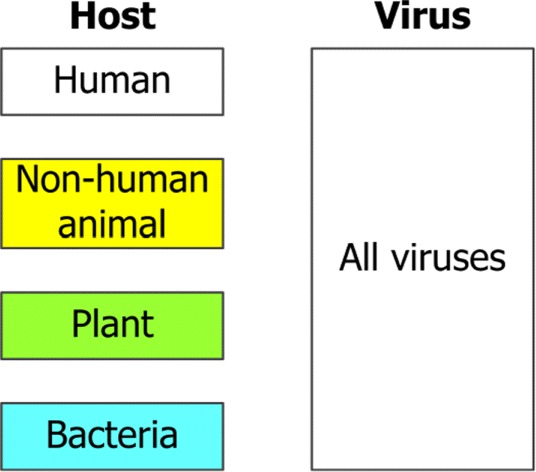
Training dataset 5 (TR5): 11,491 PPIs between human and any virus. Test dataset 5.1 (TS5.1): 488 PPIs between non-human animal and any virus. Test dataset 5.2 (TS5.2): 17 PPIs between plant and any virus. Test dataset 5.3 (TS5.3): 143 PPIs between bacteria and any virus. Test dataset 5.4 (TS5.4): 666 PPIs between non-human host and any virus (combined set of test datasets 5.1, 5.2, 5.3 and 18 PPIs with 15 other hosts)

The applicability of the prediction method to new hosts was evaluated using training dataset TR5 and test datasets TS5.1–TS5.4. TR5 contains 11,491 PPIs between human and any virus. The prediction method trained with TR5 was tested on PPIs of non-human hosts with virus, which were not used in training the method. The test datasets include TS5.1 (PPIs of non-human animal with virus), TS5.2 (PPIs of plant with virus), TS5.3 (PPIs of bacteria with virus) and TS5.4 (PPIs of any non-human host with virus) (Fig. 2).

To assess the independence of the test data from the training data, we analyzed the sequence similarity between the training datasets and test datasets using EMBOSS Needle tool [8]. As shown in Table 2, target proteins in the test datasets showed a very low sequence similarity with proteins in the training datasets (see the supporting data at http://bclab.inha.ac.kr/VirusHostPPI for the similarity of every sequence pair between the training datasets and test datasets).

**Table 2:** The average sequence similarity between proteins in training datasets and those in test datasets

		Average
Proteins in training datasets	Target proteins in test datasets	sequence
		similarity
766 virus proteins in TR1,TR3	11 H1N1 virus proteins in TS1	9.6%
774 virus proteins in TR2,TR4	3 Ebola virus proteins in TS2	10.9%
3,924 human proteins in TR5	368 non-human animal proteins in TS5.1	10.7%
3,924 human proteins in TR5	13 plant proteins in TS5.2	10.6%
3,924 human proteins in TR5	106 bacteria proteins in TS5.3	10.4%

### Features and representation

Feature selection and representation are critical to the success of prediction of PPIs. In particular, one of the challenges in sequence-based prediction of virus-host PPIs is to represent two types of proteins of variable lengths into a feature vector of a fixed length. Several encoding schemes have been used to represent protein sequences for predicting PPIs. For instance, Shen et al. [9] clustered 20 amino acids into seven groups, and represented the relative frequency of three consecutive amino acids (referred to ’amino acid triplet’) in a protein sequence using the classification. In our previous work [5], we redefined the relative frequency of an amino acid triplet using six groups of amino acids. However, both Shen’s representation and ours generate a feature vector with many zero-valued elements, which lower the prediction performance.

In this study, we represent six different features of a protein sequence in a feature vector. For representation, we fist clustered twenty amino acids into seven groups, {AGV}, {C}, {FILP}, {MSTY}, {HNQW}, {DE}, and {KR} based on the dipoles and volumes of the side chains of amino acids. The classification of amino acids is the same as that of Shen et al. [9] and others [10]. In this classification of amino acids, there are 7×7×7=343 possible amino acid triplets.

For each pair of host and virus proteins, we represent the relative frequency of amino acid triplets (RFAT) as a feature vector with 686 elements (343 for a host protein and 343 for a virus protein). The RFAT of the *i*-th amino acid triplet is defined by Eq. 1. In the equation, *f*_*i*_, *avgF*, and *maxF* denote the frequency of the *i*-th amino acid triplet, the average, and the maximum frequency of amino acid triplets in the protein sequence, respectively.

1 $$ \qquad\quad RFAT_{i} = e^{\left(f_{i}-avgF\right)\ /\ \left(maxF-avgF\right)}\\  $$

$$\begin{aligned} \text{where}\quad {avgF} &= {avg} \left\{f_{1},f_{2},\ldots,f_{343}\right\}\\ {maxF} &= {max}\left\{f_{1},f_{2},\ldots,f_{343}\right\}\\ \end{aligned} $$

Another feature is the frequency difference of amino acid triplets (FDAT) between virus and host proteins, which is defined by Eq. 2. In Eq. 2, *f*_*hi*_ is the frequency of the *i*-th amino acid triplet in the host protein of the host-virus pair, and *f*_*vi*_ is the frequency of the *i*-th amino acid triplet in the virus protein of the same host-virus pair. *avgFD* and *maxFD* denote the average and the maximum frequency difference of amino acid triplets in a host-virus pair, respectively.

2 $$\begin{array}{*{20}l} FDAT_{i} &= e^{\left(|f_{hi}-f_{vi}|-avgFD\right)\ /\ \left(maxFD-avgFD\right)}\\ \text{where}\quad {avgFD} &= {avg}\left\{|f_{h1}-f_{v1}|,\ldots,|f_{h343}-f_{v343}|\right\}\\ \textit{maxFD} &= {max} \left\{|f_{h1}-f_{v1}|,\ldots,|f_{h343}-f_{v343}|\right\} \end{array} $$

We also represent amino acid composition (AC) in each pair of host and virus proteins (Eq. 3). AC _*i*_ is the frequency of the *i*-th amino acid present in a host-virus pair divided by the maximum frequency of an amino acid in the pair.

3 $$ \qquad\qquad\quad\ \ AC_{i} \quad=\quad {\frac{f_{i}}{max \left\{{f_{1},f_{2},\ldots,f_{20}}\right\}}}  $$

The above three features, RFAT, FDAT and AC were developed in our previous study for inter-species PPIs of a single type [11]. However, the previous study used a different classification of amino acids and computed the average and the maximum frequency from all proteins in a dataset instead of a single protein being encoded.

**Fig. 3:**
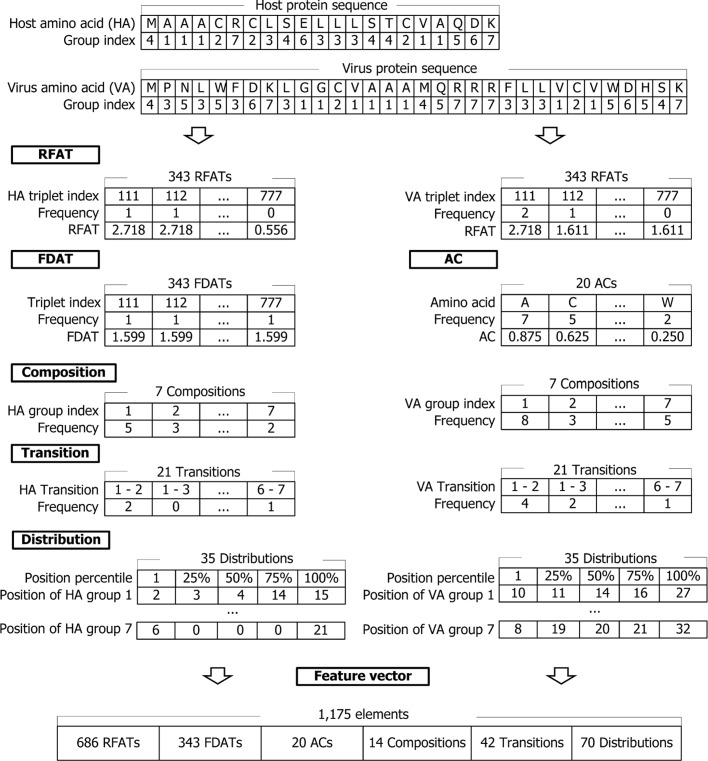
An example of a feature vector for a pair of host and virus proteins. RFAT: relative frequency of amino acid triplets. FDAT: frequency difference of amino acid triplets between virus and host proteins. AC: amino acid composition. A pair of host and virus proteins is represented by a feature vector with 1175 elements

As additional features, we used composition, transition and distribution of amino acid groups [10]. Composition represents the normalized frequency of each amino acid group in the protein sequence. Transition represents the normalized frequency of transition between each amino acid group in the protein sequence. Distribution is the normalized position of the first, 25%, 50%, 75% and 100%-th amino acid of each amino acid group in the protein sequence. A pair of host and virus proteins is represented by a feature vector with 1,175 elements (686 for RFAT, 343 for FDAT, 20 for AC, 14 for compositions, 42 for transitions, and 70 for distributions). Figure 3 shows an example of a feature vector for a pair of host and virus proteins.

## Results and discussion

### Prediction models of virus-host PPIs

We built several support vector machine (SVM) models using LIBSVM [12] to predict the interactions between virus and host proteins. The radial basis function (RBF) was used as a kernel function for training the SVM models, and the best values of parameters C and *γ* were found by running the grid search of LIBSVM on training datasets. Unless specified otherwise, the results shown in this paper were obtained with C = 32, *γ*=0.03125. The SVM models take a pair of virus and host protein sequences as input. As output, the SVM models classify whether or not the virus protein interacts with the host protein. The SVM models and supporting data are available at http://bclab.inha.ac.kr/VirusHostPPI.

### Performance measures

The performance of the prediction models were evaluated by several measures: sensitivity, specificity, accuracy, positive predictive value (PPV), negative predictive value (NPV), Matthews correlation coefficient (MCC) and the area under the ROC curve (AUC), which are defined as follows

4 $$\begin{array}{*{20}l} Sensitivity = \frac{TP}{TP+FN}\end{array} $$

5 $$\begin{array}{*{20}l} Specificity = \frac{TN}{TN+FP} \end{array} $$

6 $$\begin{array}{*{20}l} {} Accuracy = \frac{TP+TN}{TP+FP+TN+FN} \end{array} $$

7 $$\begin{array}{*{20}l} {} PPV = \frac{TP}{TP+FP}\end{array} $$

8 $$\begin{array}{*{20}l} {} NPV = \frac{TN}{TN+FN}\end{array} $$

9 $$\begin{array}{*{20}l} {} MCC = \frac{(TP\times TN)-(FP\times FN)}{\sqrt{(TP+FP)(TP+FN)(TN+FP)(TN+FN)}} \end{array} $$

In Eqs. 4–9 true positives (TP) are host proteins that are correctly predicted as interacting with a virus protein. True negatives (TN) are non-interacting host proteins that are correctly predicted as non-interacting with a virus protein. False positives (FP) are non-interacting host proteins that are incorrectly predicted as interacting with a virus protein. False negatives (FN) are interacting host proteins that are incorrectly predicted as non-interacting with a virus protein.

### Results of cross validation

We performed 10-fold cross validation of the SVM model with several datasets which contain different ratios of positive to negative data (1:1, 1:2 and 1:3). Due to the randomness of selecting negative data, we constructed three different datasets for each ratio of positive to negative data. Table 3 shows the results of the cross validation. The best performance of the SVM model was observed in the balanced dataset with 1:1 ratio of positive to negative data. As expected, running the SVM model on unbalanced datasets resulted in lower performances than running it on the balanced dataset with 1:1 ratio of positive to negative data. Datasets are available at http://bclab.inha.ac.kr/VirusHostPPI.

**Table 3:** Results of 10–fold cross validation of SVM model on 12,157 PPIs between any host-virus PPIs with different ratios of positive to negative instances

P:N	Dataset	SN(%)	SP(%)	ACC(%)	PPV(%)	NPV(%)	MCC	AUC
	1	84.93	86.03	85.48	85.87	85.09	0.709	0.926
1:1	2	84.92	86.06	85.49	85.89	85.09	0.701	0.926
	3	85.36	85.92	85.64	85.84	85.44	0.712	0.925
	mean ± SD	85.07 ± 0.3	86.00 ± 0.1	85.54 ± 0.1	85.87 ± 0.0	85.21 ± 0.2	0.71 ± 0.0	0.93 ± 0.0
	1	78.91	91.17	87.08	81.72	89.64	0.707	0.923
1:2	2	78.29	91.03	86.78	81.36	89.34	0.700	0.921
	3	78.22	91.18	86.86	81.59	89.33	0.701	0.920
	mean ± SD	78.47 ± 0.4	91.13 ± 0.1	86.91 ± 0.2	81.56 ± 0.2	89.44 ± 0.2	0.70 ± 0.0	0.92 ± 0.0
	1	74.55	93.32	88.63	78.82	91.66	0.691	0.920
1:3	2	74.61	93.56	88.82	79.43	91.70	0.696	0.919
	3	74.62	93.41	88.72	79.07	91.69	0.693	0.920
	mean ± SD	74.59 ± 0.0	93.43 ± 0.1	88.72 ± 0.1	79.11 ± 0.3	91.68 ± 0.0	0.69 ± 0.0	0.92 ± 0.0

SN: sensitivity, SP: specificity, ACC: accuracy, PPV: positive predictive value, NPV: negative predictive value, MCC: Matthews correlation coefficient, AUC: the area under the ROC

We also examined the contribution of features to the prediction performance of our SVM model. Table 4 compares different combinations of features in 10-fold cross validation of the SVM model with the 1:1 dataset of Table 3. Among the single features, RFAT was better than the others (i.e., FDAT, AC, composition, transition, and distribution) in all performance measures. With RFAT alone, the SVM model achieved an accuracy above 83% and an MCC above 0.668, which indicates that RFAT is a very powerful feature in predicting virus-host PPIs. Although RFAT is a powerful feature, performance gain was obtained with it was used with combination of other features. For example, using three features of RFAT, FDAT and AC showed a better performance than using RFAT alone. The best performance of the SVM model was observed when all six features were used.

**Table 4:** Results of 10-fold cross validation with datasets of virus-host PPIs using different combinations of features

Features	SN(%)	SP(%)	ACC(%)	PPV(%)	NPV(%)	MCC	AUC
RFAT	82.85	84.04	83.45	83.84	83.05	0.668	0.903
FDAT	68.34	57.84	63.11	61.86	64.65	0.264	0.689
AC	59.85	68.11	63.98	65.24	62.92	0.281	0.698
Composition	71.79	55.79	63.79	61.89	66.42	0.279	0.685
Transition	74.05	55.72	64.88	62.58	68.23	0.302	0.713
Distribution	71.79	31.55	51.67	51.19	52.80	0.036	0.515
RFAT+FDAT+AC	84.73	85.62	85.18	85.49	84.86	0.703	0.920
Composition+Transition +Distribution	76.51	61.72	69.12	66.65	72.43	0.386	0.787
All 6 features	85.36	85.92	85.64	85.84	85.44	0.712	0.925

SN: sensitivity, SP: specificity, ACC: accuracy, PPV: positive predictive value, NPV: negative predictive value, MCC: Matthews correlation coefficient, AUC: area under the ROC

### Applying the prediction model to new viruses

Table 5 shows the results of testing the prediction model on 2 independent datasets of PPIs of H1N1 and Ebola virus, which were not used in training the models. As discussed earlier, proteins of H1N1 virus have a sequence similarity of 9.6% to those of other viruses, and proteins of Ebola virus have a sequence similarity of 10.9% to other viruses on average. Despite such a low sequence similarity of proteins in test datasets to those in training datasets, all prediction models trained with TR1–TR4 showed a relatively high performance in independent testing. Prediction models trained with host-virus PPIs (TR2 and TR4) showed a slightly better performance than those trained with human-virus PPIs (TR1 and TR3) in both H1N1 and Ebola viruses. The models showed a higher sensitivity for Ebola virus than for H1N1 virus. Detailed information is available at http://bclab.inha.ac.kr/VirusHostPPI.

**Table 5:** Results of testing the prediction model on PPIs of new viruses

Dataset	SN(%)	SP(%)	ACC(%)	PPV(%)	NPV(%)	MCC	AUC
TR1–TS1	89.76	66.14	77.95	72.61	86.60	0.575	0.886
TR2–TS2	90.67	65.33	78.00	72.34	87.50	0.579	0.867
TR3–TS1	88.98	65.88	77.43	72.28	85.67	0.564	0.884
TR4–TS2	94.67	68.67	81.67	75.13	92.79	0.656	0.890

TR1: training dataset of PPIs between human and any virus except H1N1. TS1: test dataset of PPIs between human and H1N1 virus. TR2: training dataset of PPIs between human and any virus except Ebola virus. TS2: test dataset of PPIs between human and Ebola virus. TR3: training dataset of PPIs between any host and any virus except H1N1. TR4: training dataset of PPIs between any host and any virus except Ebola virus. SN: sensitivity, SP: specificity, ACC: accuracy, PPV: positive predictive value, NPV: negative predictive value, MCC: Matthews correlation coefficient, AUC: area under the ROC

### Applying the prediction model to new hosts

In order to examine the applicability of our prediction model to new hosts, we tested it on PPIs of viruses with new hosts, which were not used in training the model. As described earlier, the model trained with human-virus PPIs was tested on PPIs of viruses with non-human (i.e., non-human animal, plant and bacteria). As shown earlier in Table 2, the average sequence similarity of human proteins to non-human animal, plant, and bacteria is 10.7%, 10.6%, and 10.4%, respectively. Despite the low sequence similarity, tests of the model on new hosts showed a reasonable good performance (Table 6), but its performance for new hosts was slightly lower than that for new viruses.

**Table 6:** Results of testing the prediction models trained with human-virus PPIs (TR5) on PPIs of new hosts

Dataset	SN(%)	SP(%)	ACC(%)	PPV(%)	NPV(%)	MCC	AUC
TR5–TS5.1	66.39	65.98	66.19	66.12	66.26	0.324	0.733
TR5–TS5.2	76.47	58.82	67.65	65.00	71.43	0.359	0.761
TR5–TS5.3	59.44	74.83	67.13	70.25	64.85	0.347	0.736
TR5–TS5.4	64.87	67.87	66.37	66.87	65.89	0.327	0.731

TS5.1: test dataset of PPIs between non-human animal and any virus. TS5.2: test dataset of PPIs between plant and any virus. TS5.3: test dataset of PPIs between bacteria and any virus. TS5.4: test dataset of PPIs between any non-human host (non-human animal, plant, bacteria and 15 other hosts) and any virus. SN: sensitivity, SP: specificity, ACC: accuracy, PPV: positive predictive value, NPV: negative predictive value, MCC: Matthews correlation coefficient, AUC: the area under the ROC

The difference seems ascribed to the difference in the number of target proteins in test datasets and to the difference in the number of partner proteins of the target proteins, which are shared by training and test datasets. Test datasets TS1 and TS2 have 381 interactions of 11 H1N1 virus proteins and 150 interactions of 3 Ebola virus proteins with human proteins, respectively (Fig. 1 and Table 2). Test datasets TS5.1, TS5.2 and TS5.3 have 488 interactions of 368 non-human animal proteins, 17 interactions of 13 plant proteins and 143 interactions of 106 bacteria proteins with virus proteins, respectively (Fig. 2 and Table 2).

On average, a test dataset for new viruses has (381+150)/2=266 PPIs and a test dataset for new hosts has (488+17+143)/3=216 PPIs. Thus, the difference in the average number of PPIs of the two types of test datasets is not large. However, there is a big difference in the number of target proteins in the test datasets and in the number of proteins common to training and test datasets. The average number of virus proteins in a test dataset for new viruses is only (11+3)/2=7, whereas the average number of host proteins in the test datasets for new hosts is (368+13+106)/3=162. Thus, virus-host PPIs in the test datasets for new viruses share many host proteins in the training datasets (248 host proteins common to TR1 and TS1, 129 host proteins common to TR2 and TS2, 248 host proteins common to TR3 and TS1, and 129 host proteins common to TR4 and TS2) even though no virus proteins are shared by the test and the training datasets. In contrast, virus-host PPIs in the test datasets for new hosts share a much smaller number of virus proteins in the training datasets (85 virus proteins common to TR5 and TS5.1, 0 common to TR5 and TS5.2, 2 virus proteins common to TR5 and TS5.3, and 87 virus proteins common to TR5 and TS5.4).

This is a known problem with pair-input methods, which was first reported by Park and Marcotte [13], but not widely known to researchers. According to their study [13], prediction methods that operate on pairs of objects such as PPIs perform much better for test pairs that share components with a training set than for those that do not. Thus, our prediction model showed a better performance in testing for new viruses which share more partner proteins (i.e., host proteins) with training datasets than in testing for new hosts which share fewer partner proteins (i.e., virus proteins) with training datasets.

### Comparison to other methods

We compared our method with two other methods, DeNovo [6] and Barman’s method [14], using their datasets. For comparison with DeNovo’s SVM model, we tested our SVM model on DeNovo’s SLiM testing set, which contains 425 positive and 425 negative PPIs (Supplementary file S12 used in DeNovo’s study ST6). While DeNovo’s SVM model showed an accuracy of 81.90%, sensitivity of 80.71%, specificity of 83.06%, our SVM model achieved an accuracy of 84.47%, sensitivity of 80.00%, and specificity of 88.94% (Table 7). Our model showed a slightly lower sensitivity, but showed a higher specificity and accuracy. The dataset used for comparison of our SVM model with DeNovo is available at http://bclab.inha.ac.kr/VirusHostPPI.

**Table 7:** Results of testing our SVM and DeNovo’s SVM [6] on DeNovo’s dataset of 425 positive and 425 negative PPIs

	SN(%)	SP(%)	ACC(%)	PPV(%)	NPV(%)	MCC	AUC
Our SVM	80.00	88.94	84.47	87.86	81.64	0.692	0.897
DeNovo’s SVM	80.71	83.06	81.90	–	–	–	–

SN: sensitivity, SP: specificity, ACC: accuracy, PPV: positive predictive value, NPV: negative predictive value, MCC: Matthews correlation coefficient, AUC: the area under the ROC, “–”: not available

In Barman’s study [14] three machine learning methods (SVM, Naïve Bayes, and Random Forest) were used to predict virus–host PPIs using several features such as domain–domain association in interacting protein pairs and composition of methionine, serine, and valine in viral proteins. In a 5-fold cross validation with virus–host PPIs from VirusMINT [15], their SVM showed higher sensitivity and F1 score than Naïve Bayes and Random Forest. Thus, we tested our SVM model on the same dataset used in Barman’s study, which contains 1035 positive and 1,035 negative interactions between 160 virus proteins of 65 types and 667 human proteins. As shown in Table 8, our SVM model outperformed Barman’s SVM model in all performance measures. The dataset used for comparison of our SVM model with Barman’s SVM model is available at http://bclab.inha.ac.kr/VirusHostPPI.

**Table 8:** Results of 5-fold cross validation of our SVM and Barman’s SVM [14] with Barman’s dataset of 1035 positive and 1035 negative PPIs

	SN(%)	SP(%)	ACC(%)	PPV(%)	NPV(%)	MCC	AUC	F1(%)
Our SVM	76.14	83.77	79.95	82.46	77.80	0.601	0.858	79.17
Barman’s SVM	67.00	74.00	71.00	72.00	–	0.440	0.730	69.41

SN: sensitivity, SP: specificity, ACC: accuracy, PPV: positive predictive value, NPV: negative predictive value, MCC: Matthews correlation coefficient, AUC: the area under the ROC, F1 = 2x(SNxPPV)/(SN+PPV), “–”: not available

## Conclusion

Most computational methods of predicting PPIs are intended for interactions within a species rather than for interactions across different species such as interactions between virus and host cell proteins. A small number of computational methods which were recently developed for predicting PPIs between virus and host are limited to interactions of single virus or single host, and therefore a separate prediction model is required to predict PPIs of new viruses or hosts. However, proteins of new viruses or hosts often exhibit quite a low sequence similarity to proteins of known viruses or hosts, and little information is available for new viruses or hosts.

In this study, we developed a prediction model of virus-host PPIs, which is applicable to new viruses and hosts. We tested the prediction model on independent datasets of virus-host PPIs, which were not used in training the model and have a very low sequence similarity to any protein in training datasets of the model. Despite a low sequence similarity between proteins in training datasets and target proteins in test datasets, the prediction model showed a high performance comparable to the best performance of other methods for single virus-host PPIs. Our prediction model will be useful in finding potential PPIs of new viruses with new hosts, for which little information is known.
